# Predator Diversity and Abundance Provide Little Support for the Enemies Hypothesis in Forests of High Tree Diversity

**DOI:** 10.1371/journal.pone.0022905

**Published:** 2011-07-28

**Authors:** Andreas Schuldt, Sabine Both, Helge Bruelheide, Werner Härdtle, Bernhard Schmid, Hongzhang Zhou, Thorsten Assmann

**Affiliations:** 1 Institute of Ecology, Leuphana University Lüneburg, Lüneburg, Germany; 2 Institute of Biology/Geobotany and Botanical Garden, Martin-Luther-University Halle-Wittenberg, Halle, Germany; 3 Institute of Evolutionary Biology and Environmental Sciences, University of Zurich, Zurich, Switzerland; 4 Institute of Zoology, Chinese Academy of Sciences, Beijing, China; University of Alberta, Canada

## Abstract

Predatory arthropods can exert strong top-down control on ecosystem functions. However, despite extensive theory and experimental manipulations of predator diversity, our knowledge about relationships between plant and predator diversity—and thus information on the relevance of experimental findings—for species-rich, natural ecosystems is limited. We studied activity abundance and species richness of epigeic spiders in a highly diverse forest ecosystem in subtropical China across 27 forest stands which formed a gradient in tree diversity of 25–69 species per plot. The enemies hypothesis predicts higher predator abundance and diversity, and concomitantly more effective top-down control of food webs, with increasing plant diversity. However, in our study, activity abundance and observed species richness of spiders decreased with increasing tree species richness. There was only a weak, non-significant relationship with tree richness when spider richness was rarefied, i.e. corrected for different total abundances of spiders. Only foraging guild richness (i.e. the diversity of hunting modes) of spiders was positively related to tree species richness. Plant species richness in the herb layer had no significant effects on spiders. Our results thus provide little support for the enemies hypothesis—derived from studies in less diverse ecosystems—of a positive relationship between predator and plant diversity. Our findings for an important group of generalist predators question whether stronger top-down control of food webs can be expected in the more plant diverse stands of our forest ecosystem. Biotic interactions could play important roles in mediating the observed relationships between spider and plant diversity, but further testing is required for a more detailed mechanistic understanding. Our findings have implications for evaluating the way in which theoretical predictions and experimental findings of functional predator effects apply to species-rich forest ecosystems, in which trophic interactions are often considered to be of crucial importance for the maintenance of high plant diversity.

## Introduction

The presence, abundance and biodiversity of predatory arthropods have significant impacts on the functioning of ecosystems [1]–[4]. Predator-mediated changes in herbivore feeding preferences or intensity can alter plant community structure and diversity (e.g. [5]). Interactions between predators and detritivores affect decomposition dynamics [6]. While the importance of these trophic interactions in influencing and modifying ecosystem processes such as biomass production and nutrient cycling is increasingly recognized, and trophic complexity is increasingly being implemented in ecosystem functioning experiments [1], [7], [8], our basic knowledge on how the diversity and abundance of secondary consumers relates to plant diversity in natural ecosystems is still limited [9]–[11]. More information on the relationship between the biodiversity at different trophic levels is required to understand how natural ecosystems and their functioning are influenced by the potentially diversity-dependent effects of trophic interactions reported from experiments or theory [8], [12]. This knowledge is also of crucial importance in biodiversity conservation (e.g. [13]).

Generally, predator abundance and diversity are expected to increase with increasing plant diversity, as diverse plant communities are hypothesized to offer a greater amount of resources (in terms of biomass production and resource heterogeneity; [14]–[16]) to consumers. A popular hypothesis concerned with trophic interactions in relation to species diversity is the ‘enemies hypothesis’ [17], which predicts that predators are more abundant and more diverse (and can thus more effectively regulate lower trophic levels such as herbivores) in species-rich plant communities because these communities offer a greater variety of habitats as well as a broader spectrum and temporally more stable availability of prey [18]. Many of the studies which analyzed predator diversity and abundance in relation to plant diversity so far, however, only compared monocultures to mixtures of few plant species (e.g. [11], [19], [20]). Results of these studies were ambiguous and often depended on the plant species studied, with strong effects of plant species identity making it difficult to assess the effect of plant species richness *per se* [21]–[23]. Several studies in grassland ecosystems have also analyzed the relationship between plant diversity and predators over larger gradients of plant diversity, but here again results were mixed [10], [24]–[27]. For more complex ecosystems such as forests, however, which are characterized by long-lived plant individuals and which provide critically important ecosystem services [28], comparable studies including high diversity levels are lacking [11]. Yet, species-rich forests are of particular interest in this respect, as trophic interactions might play an important role in maintaining the high levels of tree diversity in these ecosystems [29]–[31].

Here, we analyze activity abundance and species richness of an important group of predatory forest arthropods, epigeic spiders, across 27 differentially diverse forest stands (between 25 and 69 tree and shrub species per 900 m^2^; [32]) of different ages in subtropical China. Epigeic arthropods make up a large part of the overall faunal diversity in plant species-rich forests [33] and can play an indirect role in the long-term maintenance of tree diversity: ground-active predators can particularly affect densities of insect herbivores feeding on recruits (seedlings and saplings) growing close to the forest floor (e.g. [34]), i.e., on plant individuals which will determine tree diversity in the long run. These predators might even affect herbivores of higher vegetation strata, as many of these herbivores develop or take shelter during inactivity periods on the forest floor [11], [22], [35], [36]. Moreover, the diversity and abundance of epigeic predators can strongly affect decomposer assemblages and thus influence ecosystem functions such as nutrient cycling [6]. Tree species diversity, in turn, can directly or indirectly feed back on epigeic arthropods by affecting abiotic and biotic characteristics (e.g. litter depth and structure, microclimate, pH, prey availability and vegetation structure) of the forest floor [37], [38]. We tested to which degree predator assemblages at plant diversity levels beyond the scope of most previous biodiversity studies respond to differences in plant diversity. Whether relationships observed at lower levels of plant diversity reach an asymptote at higher diversity or not is still unclear [39], [40]. Epigeic spiders might respond positively to higher structural heterogeneity (e.g., via a more diverse litter layer or a potentially higher herb layer diversity) and potentially increased prey availability in forest stands of high tree diversity, which would be in accordance with the enemies hypothesis [16], [18]. Interestingly, in a previous study we found that insect herbivory on saplings (i.e., tree individuals with a strong connection to the forest floor) in these 27 study plots was higher in the more diverse plant stands [41]: this is in contrast to the predictions of the enemies hypothesis and more consistent with a positive bottom-up effect of plant diversity on herbivore diversity [42]. Our present study provides information needed for a better understanding of the role of trophic interactions in the long-term maintenance of high plant diversity and the functioning of such phytodiverse ecosystems [10], [29], [43] by testing one of the major assumptions of the enemies hypothesis (increasing abundance and richness of predators with higher plant species richness) for an important group of generalist predators.

## Methods

### Study site and plot selection

The study was conducted in the Gutianshan National Nature Reserve (GNNR; 29°14′ N, 118°07′ E), Zhejiang Province, in South-East China. The GNNR is located in a mountain range at an elevation of 300–1260 m a.s.l. It was established as a National Forest Reserve in 1975 and is characterized by 8000 ha of semi-evergreen, broad-leaved forests in a subtropical monsoon climate. The mean annual temperature is 15.3°C; mean annual precipitation amounts to ca. 2000 mm. The parent rock of the mountain range is granite, with pH ranging from 5.5–6.5 [44], [45].

Within the framework of the ‘BEF China’ project [32], we established 27 study plots of 30×30 m in the GNNR. The original intention was to select plots according to a factorial design of three richness levels of woody species and three successional stages. However, it was not possible to find young stages with high richness. Thus, the plots, which represented a deliberately large range of woody species richness (25–69 tree and shrub species per plot), were stratified a posteriori according to stand age (between <20 and ≥80 years, [32]) into five classes reflecting different successional stages and woody species richness. Plots were randomly distributed throughout the reserve, with limitations due to inaccessibility or inclinations >55°. Typical tree species of this subtropical forest are the evergreen *Castanopsis eyrei* (Champ. ex Benth.) Tutch and *Schima superba* Gardn. et Champ. Mean height of the upper tree layer varies from 13–25 m along the successional gradient. Further details on plot establishment and plot characteristics are provided in [32].

### Spider data

In each of the 27 study plots, four pitfall traps (i.e. a total of 108 traps) were installed for standardized trapping of epigeic arthropods. The traps were set up at the corners of a 10×10 m square around the center of each plot and consisted of a plastic cup (diameter 8.5 cm, depth 15 cm, capacity 550 ml) sunk into the ground and filled with 150 ml of preserving solution (40% ethanol, 30% water, 20% glycerol, 10% acetic acid, with a few drops of detergent to reduce surface tension) for continuous trapping. Sampling was conducted in 2009 for five months (30 March–2 September) and covered the main growing season. The traps were emptied and refilled at 14 day intervals. As composite measures of activity and abundance of the species caught [46], pitfall traps record ‘activity abundances’. These can be interpreted as longer-term (over the trapping interval) patterns in the locomotory activity and the densities of individual species [47]. In the following, we use ‘activity abundance’ to characterize the trap catches.

Spiders were sorted and determined to species or morphospecies level. Classification of spiders by morphospecies (within families or genera) can be easily and reliably achieved on the basis of their genitalia (e.g. [48]). For our analyses we further assigned spiders to foraging guilds. The functional effects of spiders depend on their foraging mode, and foraging guild diversity can thus be a functionally important characteristic of spider assemblages [5], [49]. Guild classification was based on the primary hunting mode of the respective family ([50], [51] and own observations) and comprised the following nine guilds: orb-web weavers (Araneidae, Tetragnathidae), space-web weavers (Dictynidae, Theridiidae), sheet-web weavers (Hahniidae, Linyphiidae), ground-funnel-web weavers (Agelenidae, Amaurobiidae), ground-space-web weavers (Leptonetidae), ground-tube-web or burrow weavers (Atypidae, Ctenizidae, Hexathelidae, Nemesiidae), trip-line-retreat builders (Segestriidae), foliage hunters (Clubionidae, Ctenidae, Mimetidae, Philodromidae, Pisauridae, Salticidae, Sparassidae, Thomisidae) and ground hunters (Corinnidae, Gnaphosidae, Liocranidae, Lycosidae, Oonopidae, Zodariidae, Zoridae).

While scales of perception of plant diversity by epigeic spiders might vary between species, this does not affect our results: first, our plots represent subsections of larger forest expanses for which they could be considered typical; second, many predatory arthropods can establish viable populations already in areas as small as our study plots (e.g. [52]); third, woody plant species richness at the plot level was also highly correlated with plant species richness at subplot levels (Pearson's *r* between 0.88 and 0.72 for correlations between total and rarefied richness for 200–20 tree individuals [41]).

### Environmental predictors

Observational studies allow for the analysis of ecological patterns and processes under near-natural conditions (e.g. fully established animal and plant communities) in complex, real-world ecosystems [53]. However, adequate interpretation of species richness effects in such studies requires that potentially confounding environmental factors, which might be correlated with plant species richness and might directly or indirectly affect spiders, are taken into consideration [54]. We thus included a set of environmental variables in the analyses to account for potential effects of important abiotic (e.g. soil pH, vegetation-mediated light availability) and biotic (e.g. plant biomass, which might, for instance, affect prey densities) characteristics of the plots and the immediate surroundings of the traps: besides successional stage and species richness of woody plants (see above), canopy and herb cover, altitude, tree density (all tree and shrub individuals >1 m height—constituting the bulk of plant biomass and production in the plots) were assessed for all plots during plot establishment in 2008. Total basal area of trees and shrubs as a measure of plot biomass was calculated from diameter at breast height (dbh) measurements of all trees >10 cm dbh in the whole plot and for all individuals >3 cm dbh in a central subplot of 10×10 m. The pH of the topsoil (0–5 cm) was determined from nine dried and sieved soil samples per plot, taken in the summer of 2009. These were pooled and measured potentiometrically in a water-soil solution [32]. To take into account differences in the surrounding matrix of the pitfall traps, which can affect spider movement and catch efficiency [46], [47], we further recorded litter depth, percentage cover of litter and of plants in the (in many cases relatively sparsely developed) herb layer, and vegetation height of the herb layer in a 1×1 m grid around each trap in the summer of 2009. We also included the richness of plant species in the herb layer (all plant individuals <1 m height, measured in the 10×10 m central subplot) to distinguish between effects of the tree (e.g. via tree litter heterogeneity) and the herb layer (i.e., horizontal plant structure within the realm of ground-active spiders).

### Statistical analysis

All analyses were performed using R 2.8.1 [55]. Activity abundance was square-root transformed to meet assumptions of normality and homogeneity of variances. Spider species richness in our samples was found to be correlated with spider activity abundance (Pearson's *r* = 0.63; *P*<0.001). We thus used two different measures of spider species richness, observed and individual-based rarefied, to analyze relationships between the richness of woody plant species and spider species. Rarefaction calculates species numbers for a standardized number of individuals across all samples and yields species richness data which are independent of the number of individuals in a particular sample, as the latter is potentially affected by differences in sampling efficiency. However, differences in the number of individuals sampled may also reflect real and biologically meaningful patterns [56]. Thus, the two measures allow for a simultaneous assessment of pure (rarefied richness) and abundance-mediated (observed richness) responses of spider species richness to differences in woody plant species richness. We also checked for the completeness of our trap catches with nonparametric first-order jackknife estimation [57]. Rarefaction and species estimations were performed using the package vegan [58]. Species richness of woody plants was not affected by potential sampling bias and thus not corrected for differences in the total number of individuals per plot (density). Furthermore, observed and rarefied (for n = 200 individual plants) species richness of woody plants were highly correlated (*r* = 0.88; *P*<0.001) because observed species richness was not correlated with density of woody plants (*r* = −0.07; *P* = 0.737). The species richness of woody plants was also not correlated with the abundance of any of the dominant tree or shrub species (Pearson correlations with the eight most abundant species, which accounted for >55% of all tree individuals in the 27 study plots, were all non-significant; not shown), i.e., relationships between woody plant species richness and spiders are independent of and not affected by the species identity of the dominant tree and shrub species in the individual study plots.

The relationships between a) activity abundance, b) observed spider species richness, c) rarefied spider species richness and d) foraging guild richness of spiders as response variables and species richness of woody plants as an explanatory variable were analyzed with linear mixed-effects models, using the package nlme in R [59]. Mixed-effects models take into account hierarchical structures and potential non-independence of data by the inclusion of a random effects structure [60]. In our case, the hierarchical structure was given by the traps nested within plots; thus, plot identity was fitted as random effect. We checked for significant nonlinear relationships between the response variables and the predictors by analyzing second- and third-order polynomials of the predictors. Before fitting the full model, the environmental predictors were checked for collinearity. Tree density (Pearson's *r* = −0.77; *P*<0.001) and total basal area (*r* = 0.82; *P*<0.001) were strongly related to successional stage and primarily reflected stand age-related differences in plot characteristics (see also [41]). Likewise, vegetation height of the herb layer was strongly correlated with vegetation cover around the traps (*r* = 0.73; *P*<0.001). To avoid potential effects of multicollinearity, we did not include tree density, total basal area or vegetation height in the models. The full models were thus fitted with successional stage, canopy and herb cover, altitude, soil pH, woody plant species richness of the shrub and tree layers, and the richness of plant species in the herb layer as covariates representing plot characteristics, and with litter cover, litter depth and vegetation cover as covariates representing characteristics of the microhabitat around the traps within plots. We also fitted the interaction between woody plant species richness and stand age to check whether potential species richness effects depended on the successional age of the forest stands.

We used model simplification with an information-theoretic approach to obtain the most parsimonious explanatory models. Model simplification was based on the Akaike Information Criterion, corrected for small sample sizes (AIC_c_ [61]). Predictors whose exclusion improved model fit by reducing the AIC_c_ of the resulting model were eliminated in an automated stepwise procedure (a modified version of the stepAIC procedure in R; [42]) until a minimal, best-fit model with the lowest global AIC_c_ was obtained. The model with the smallest number of predictors was chosen as being the most parsimonious in case differences in AIC_c_ (ΔAIC_c_) of ≤2 between two candidate models indicated that both models are almost equally likely [61]. Model residuals were checked for assumptions of normality and homoscedasticity. Further, we calculated Moran's I coefficients to test for spatial autocorrelation in the model residuals, using the R package ncf [62].

## Results

### Species numbers, activity abundance and foraging guild richness

In total, 7952 spiders (of which 6166 were adults), belonging to 195 (morpho-) species of 29 families, were captured in pitfall traps. The most species-rich families in the forest stands were Salticidae (35 species) and Linyphiidae (30), while the most abundant families were Lycosidae (2125 individuals belonging to 6 species) and Liocranidae (1485 individuals of 12 species). First-order jackknife estimation (with traps as samples) showed that all plots were equally sampled, with 66–78% of the estimated species numbers for each plot. In total, 268 (±19 *SE*) epigeic spider species can be expected to occur on the forest floor of the 27 study sites.

**Figure 1:**
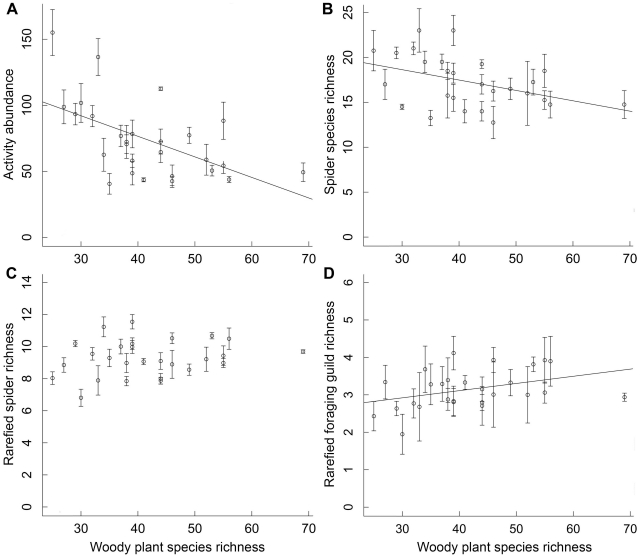
Relationships between species richness of woody plants and spiders. (A) activity abundance, (B) original species richness, (C) rarefied species richness, and (D) rarefied foraging guild richness of epigeic spiders (trap means ± SE for each plot) across a plant species diversity gradient of 27 study plots in subtropical China. Regression lines show significant relationships at *P*<0.05.

The mean number of spider individuals per trap decreased strongly from more than 100 in the plots with the lowest woody plant species richness to about 50 individuals in the plots with the highest woody plant species richness (Figure 1A), and a minimal model with negative effects of woody plant species richness (*t* = −3.8; *P*<0.001) and positive effects of soil pH (*t* = 3.0; *P* = 0.007) best explained the observed patterns in activity abundance (Table 1). There was a strong positive relationship between activity abundance and the species richness of spiders in the study plots (see Methods section), and, like abundance, mean spider richness per plot (trap means ranging from 12.8 to 23.0) decreased significantly with increasing woody plant species richness (Figure 1B). Woody plant species richness (*t* = −2.6; *P* = 0.015), together with a negative effect of altitude (*t* = −3.2; *P* = 0.004), was also retained in the minimal mixed-effects model for spider richness when potentially confounding plot characteristics were accounted for (Table 1). Species richness and activity abundance of spiders were neither affected by plant species richness of the herb layer, nor by litter cover, litter depth or vegetation cover in the immediate surroundings of the traps, and none of these variables were retained in any of the minimal mixed-effects models (Table 1).

**Table 1:** Mixed-effects models for spider species richness and activity abundance.

	Activity abundance^b^				Spider species richness				Rarefied richness				Foraging guilds (rarefied)			
*Fixed effects* a	*DF_n_*	*DF_d_*	*F* c	*P*	*DF_n_*	*DF_d_*	*F* c	*P*	*DF_n_*	*DF_d_*	*F* c	*P*	*DF_n_*	*DF_d_*	*F* c	*P*
Successional stage	-	-	-	-	-	-	-	-	4	22	2.9	0.045	-	-	-	-
Herb cover	-	-	-	-	-	-	-	-	-	-	-	-	1	24	7.9 (+)	0.009
Altitude	-	-	-	-	1	24	10.9 (−)	0.003	-	-	-	-	-	-	-	-
Soil pH	1	24	8.7 (+)	0.007	-	-	-	-	-	-	-	-	-	-	-	-
Woody plant species richness	1	24	14.5 (−)	<0.001	1	24	6.9 (−)	0.015	-	-	-	-	1	24	6.8 (+)	0.015
*AIC_c_ full model* d	396.1				598.5				371.1				199.9			
*AIC_c_ minimal model*	373.5				578.5				349.4				179.9			

Results for the fixed effects of the minimal mixed-effects models (numerator and denominator degrees of freedom DF_n_ and DF_d_; *F*-value and probabilities *P*; terms dropped during model simplification are marked “−”) for activity abundance, original and rarefied species richness, and rarefied foraging guild richness of spiders as response variables.

a Canopy cover, litter cover (trap surroundings), litter depth (trap surroundings), vegetation cover of the herb layer (trap surroundings) and interaction successional age:woody plant species richness (non-significant and excluded in all cases during model simplification) not shown.

b Square root-transformed.

c (+) and (−) indicate positive and negative relationship, respectively.

d Full model: fitted with the full set of fixed effects; minimal model: simplified model with lowest AIC_c_.

**Figure 2:**
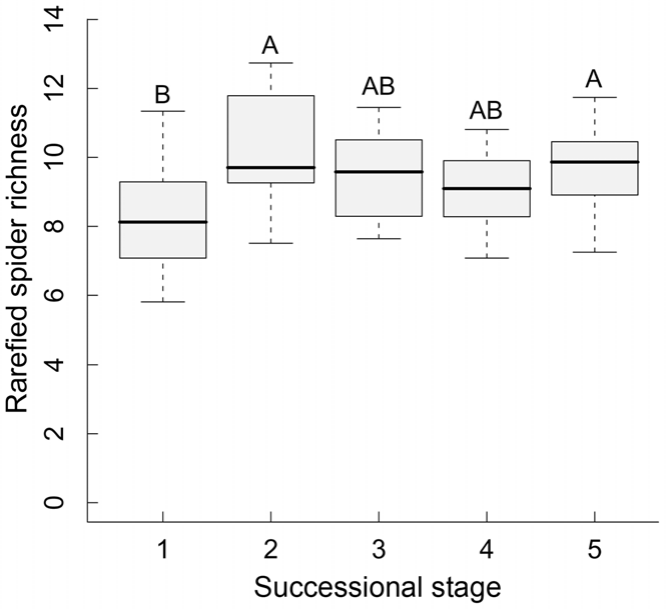
Rarefied spider species richness in relation to plot age. Mean values per trap are shown in relation to the successional stage (1–5: <20, <40, <60, <80 and ≥80 years old) of the 27 subtropical forest stands in south-east China. Different letters indicate significant differences between successional stages at *P*<0.05.

Rarefied species richness of spiders tended to increase slightly across the gradient of woody plant species richness (Figure 1C). However, this effect was not significant, and thus woody plant species richness was not retained in the minimal mixed-effects model for rarefied spider richness, which only included successional age as an explanatory variable (Table 1). Rarefied spider species richness was high in plots >20 yr and lowest in the youngest forest stands (Figure 2). In contrast, there was a significant increase in rarefied feeding-guild richness of spiders with increasing species richness of trees and shrubs (Figure 1D). The minimal mixed-effects model pointed out positive effects of both woody plant species richness (*t* = 2.6; *P* = 0.015) and herb cover (*t* = 3.0; *P* = 0.006) in the forest stands (Table 1). The results of our study were not affected by the sequence in which woody plant species richness and stand age were fitted in the analyses (i.e., results did not differ between models with plant richness fitted before or after stand age; not shown). There was no significant spatial autocorrelation in the residuals of the minimal mixed models, with Moran's I values all close to zero and *P*>0.05 (not shown).

## Discussion

The results of our study provide insight into the relationship between predator and plant diversity for complex forest ecosystems, extending our knowledge from observational and experimental studies of relatively species-poor to highly diverse forest ecosystems. Our findings for spider activity and species richness only partially reflect patterns reported from studies of species-poor forests or other ecosystems and do not unambiguously support common hypotheses on diversity-dependent relationships between predators and other trophic levels.

### Spider activity abundance

Contrary to what might have been expected, we observed a decrease in activity abundance of spiders in forest stands of high woody plant diversity. Considering the commonly stated positive plant productivity–diversity relationship (cf. [39]) and the predictions made by the enemies hypothesis [17], we would have expected to find the opposite pattern of higher predator activity abundance (and higher species richness, see below) in more diverse forest stands. This pattern was observed for predator activity and abundance in several previous studies, mainly of non-forest ecosystems (e.g. [10], [19], [63] and references therein). In contrast, results from the few studies conducted in forests were ambiguous and, due to comparisons of relatively species-poor stands, often strongly affected by tree species identity [11], [20], [23].

A negative relationship between the activity of predators and plant diversity across a gradient from low to relatively high plant species richness was also found by Koricheva et al. [24] in an experimental grassland study. They attributed this negative relationship primarily to indirect effects of plant diversity on predator activity through diversity-dependent changes in microclimate and prey availability. This probably does not apply in the same way to our study, as characteristics of the plots and the immediate trap surroundings which are often considered to influence the activity of ground-dwelling arthropods, such as vegetation density or litter depth (which, in turn, affect habitat structure and microclimatic conditions; [64], [65]), had no effect on spider activity abundance. The only abiotic variable which significantly covaried with spider activity abundance in our study was soil pH (which ranged between 4.1 and 5.1), which was not related to plant diversity. However, woody plant species richness had a stronger effect than pH and was retained in the minimal mixed-effects model. Our results thus indicate a negative effect of plant diversity on spider activity abundance independent of covarying plot characteristics. This effect is due to changes in tree- and shrub-layer, rather than herb-layer plant diversity, as the latter was not related to our spider data. This suggests that in the studied forest stands, the horizontal plant structure of the herb layer has little impact on epigeic spiders compared to the effects of the tree and shrub layers. Forming the dominant vegetation strata of the studied forests in terms of biomass, the latter layers and their plant diversity can be expected to have strong effects on abiotic (e.g. litter diversity) or biotic (e.g., faunal assemblage structure) characteristics at the forest floor. Missing effects of important abiotic parameters, in particular of litter depth and cover, on spider activity abundance, indicate that biotic characteristics mediated by tree diversity might play an important role in determining the observed patterns.

While a higher prey abundance in the more diverse forest stands could potentially reduce foraging time and thus spider activity, the opposite pattern of higher spider activity in forest stands with higher prey availability has also been reported [23], which shows that prey availability cannot be used consistently as a predictor of predator activity. It will be intriguing to further explore the potential causes of the unexpected negative relationship between spider activity abundance (and observed species richness) and tree diversity. For instance, patterns in richness and abundance of spiders could be affected by the abundance or diversity of their enemies (e.g. pompilid wasps, birds, vertebrates) or competing predatory taxa (e.g. ants) (see e.g. [66], [67]). Elucidating the mechanisms underlying the observed patterns requires further investigation. Yet, the facts that in our study plots herbivore damage levels of saplings increased with increasing species richness of woody plants [41] and that these damage levels are negatively correlated with spider activity abundance (Pearson'*s r* = −0.48; *P* = 0.012) indicate that the influence of important predator groups on herbivores is not necessarily higher in the forest stands with higher tree and shrub diversity. With seedlings and saplings growing close to the forest floor, interactions between epigeic predators and herbivores (see Introduction) can be important for these tree recruits, which play a key role in the long-term maintenance of tree diversity. The absence of positive predator effects with increasing plant species richness would be in contradiction to predictions of the enemies hypothesis (see e.g. [17], [18]) and to suggestions from a recent grassland study [10]; however, studies of less diverse forest ecosystems [11], [22] also found no evidence of the effects predicted by the enemies hypothesis, as can also be deduced from a further study on grassland systems [68].

### Species richness and foraging guilds

In contrast to predator activity and abundance, little information is available on patterns of predator species richness across gradients of high plant diversity, and this information is basically limited to non-forest ecosystems. In a long-term grassland experiment Haddad et al. [10] found that species richness of predators was positively related to plant diversity (see also [26]). However, species numbers depended on predator abundance, and rarefied species richness actually declined with increasing plant diversity. The positive effect of plant diversity on the observed species richness was attributed to higher numbers of individuals in more productive plots, in accordance with the more individuals hypothesis, which assumes that more productive sites (in terms of biomass) support larger populations of a greater number of consumer species than less productive sites [10]. We also found a strong dependence of species richness patterns of spiders on activity abundance, however, with the opposite effect of decreasing activity and richness with increasing tree species richness. Our activity abundance data do not directly allow for quantification of actual abundance patterns per unit area (cf. [46]). An evaluation of the productivity–abundance relationship as implied by the more individuals hypothesis [15] is thus not directly possible in our case, as we cannot completely exclude effects of prey availability on activity patterns. However, even with richness patterns potentially influenced by effects of prey availability on spider activity in the study plots, these patterns mean that the activity-dependent species density of spiders is lower in plots with higher tree diversity. Reduced species density can affect prey organisms such as herbivores or detritivores, as the behavior of different predator species (regarding, for instance, foraging mode and foraging intensity) influences prey behavior and performance [5], [49]. Lower species densities due to lower predator activity (see above) might thus also contribute to less strong top-down control and to effects such as higher herbivory in forest stands with high tree diversity (see also [7]). In our case, this might primarily apply to effects on seedlings and saplings, which grow close to the forest floor. However, long-term maintenance of tree diversity essentially depends on these tree recruits and thus on trophic interactions influencing tree recruitment [31], [43]. Moreover, changes in the strength of top-down control can also have effects on other important ecosystem functions, such as nutrient cycling, via predator impacts on decomposer food webs [6].

Even for rarefied species richness of spiders, which is independent of the observed spider activity abundance, our results are not supportive of the assumed positive effects of plant diversity and of the concomitant higher structural heterogeneity on the species richness of predators, as proposed by the enemies hypothesis and other related hypotheses [10], [16], [18]. Removing the effect of activity abundance on species richness of spiders resulted in the elimination of woody plant species richness as a predictor of spider species richness in the mixed model analysis. Even though a tendency towards increasing rarefied spider richness with increasing plant diversity might be discernible, this relationship was of low explanatory power and not significant for rarefied spider species richness. Instead, effects of forest stand age became important for rarefied richness. Forest age can have strong impacts on animal communities because not only biotic conditions such as plant diversity but also abiotic conditions change considerably during the course of forest succession [53], [54]. The results of our study were independent of the sequence in which woody plant richness and stand age were fitted in the analyses. When effects of the number of spider individuals are factored out, successional age thus seems to overrule effects of tree diversity on species richness of epigeic spiders in our subtropical study system.

Interestingly, woody plant species richness positively affected the rarefied number of spider foraging guilds. Higher structural heterogeneity, as also shown by a positive relationship with herb layer cover, probably promotes the coexistence of species with different foraging behavior in plots with high plant diversity (cf. [51]). In contrast to mere species numbers, results for foraging guilds as an aspect of functional diversity are in accordance with predictions of the enemies hypothesis. In general, such higher functional diversity of predators has been shown to affect ecosystem processes, as different hunting modes of spiders can strongly impact herbivore behavior (e.g. [49]). However, in view of our findings for spider activity and the herbivory patterns observed for the study sites [41], further research is needed to evaluate the significance of this increase in feeding-guild richness for trophic interactions such as herbivory, and to assess how this affects ecosystem processes in these forests (cf. [2]).

### Conclusions

Ground-dwelling arthropods make up a large part of the invertebrate biodiversity in forests of high tree diversity [33] and can have strong effects on food webs also of higher vegetation strata [11], [22], [35], [36]. Knowledge of the diversity of these invertebrates and of their interactions across trophic levels is essential for our understanding of the functioning of these ecosystems [43]. Our study provides information on predator diversity across a gradient of tree diversity far beyond the range of previous studies in forest ecosystems. For dominant epigeic predators, our results contradict common hypotheses of predator–plant diversity relationships, such as the enemies hypothesis, which were derived from studies in less diverse ecosystems. In view of previous findings of increased herbivory in the more diverse forest stands of our study sites it is questionable whether effects predicted from this hypothesis, for which support is also already mixed for less diverse ecosystems, have a strong impact on ecosystem processes also in higher vegetation strata of our subtropical forest ecosystem. Our study supports findings from previous studies of species-rich ecosystems which state that predator diversity is not necessarily a positive or simple function of plant diversity in such highly diverse plant communities [24], [25]. As our diversity gradient started at medium diversity levels it might be possible that positive effects often observed at lower plant diversity levels have leveled off (e.g. due to redundancy effects) in our forest stands (cf. [39]). Our results have implications for evaluating the way in which theoretical predictions and experimental findings of functional effects of predators apply to such ecosystems of high tree diversity, in which trophic interactions are often considered to be of crucial importance for the maintenance of high plant diversity [29]–[31]. Further exploration under experimentally controlled conditions, such as in the new tree plantations of the BEF China project, will help to shed light on the ecosystem consequences of the observed patterns.
